# The effects of physical activity on adolescents’ depression: evidence from China

**DOI:** 10.3389/fpsyg.2024.1430145

**Published:** 2024-07-30

**Authors:** Hongmei Chen, Meng Liu, Wenqing Zhao, Hanlin Wei, Ying Zhang, Shunguo Li

**Affiliations:** ^1^Institute of Millet Crops, Hebei Academy of Agriculture and Forestry Sciences, Shijiazhuang, Hebei, China; ^2^College of Economics and Management, Tarim University, Alar, Xinjiang, China; ^3^College of Economics and Management, China Agricultural University, Beijing, China; ^4^Characteristic Industry Association of Hebei Province, Hebei, China

**Keywords:** physical activity, depression, adolescents, China, CES-D scale

## Abstract

**Background:**

Depression is becoming a common threat to the mental health of Chinese adolescents. As an intermediate stage between being healthy and having depression, identifying factors influencing depressive may contribute to providing more options for the prevention and treatment of depression.

**Objective:**

The study aims to explore the effects of physical activity on adolescent depression, focusing on the times and hours of activity per week.

**Methods:**

The study used a cross-sectional dataset collected in Ruyang County, Henan Province in September 2022, including a sample of 5,629 adolescents in 31 compulsory public schools in the county. We utilized Ordinary Least Squares (OLS) to analyze the impact of physical activity on adolescents’ depression scores, and probit model to analyze the influence of physical activity on adolescents’ depression. To examine whether there is a U-shaped relationship between physical activity and depression, we included the squared terms of times and hours of activity in models.

**Results:**

The results showed that: (1) The times of physical activity significantly reduces Chinese adolescent depression. An increase in physical activity by one time per week is associated with a mean decrease of 0.354 points in depression scores (*p* < 0.01). However, an increase of one time of physical activity per week is associated with an average 1% increase in the likelihood of experiencing depression(*p* < 0.05), while the hours of physical activity was statistically insignificant. (2) Physical activity has a U-shaped (not linear) relationship with adolescent depression, with 7–8 times per week or 7–9 h of physical activity per week being the optimal range.

**Conclusion:**

The study found that increasing the frequency of physical activity positively impacts adolescent depression, while increasing the hours does not show a significant association. Furthermore, a U-shaped relationship exists between times of activity, hours of activity, and adolescent depression, suggesting that moderate activity is optimal.

## 1 Introduction

Depression is becoming a common threat to the mental health of Chinese children and adolescents (Tepper et al., 2008). This concern stems from reports indicating that depression is increasingly becoming the predominant challenge among Chinese youth (Tepper et al., 2008). A comprehensive meta-analysis, encompassing over 230,000 Chinese children and adolescents, uncovered that a range of 10.3 to 54.5% of screened adolescents exhibited positive indications of depression, signaling a rising prevalence of depressive symptoms among this demographic (Li et al., 2019). As a transitional phase between being healthy and having depression, students with subthreshold depression may either progress to more severe depression or improve with preventive intervention (Jiang et al., 2019). Therefore, identifying more influences on depression may help provide more options for the prevention and treatment of depression.

There are many factors influencing adolescent depression, including academic stress (Zhu et al., 2021), physical activity (Stanton and Reaburn, 2014), sleep (Zou et al., 2023), and obesity (Wang et al., 2022), and adolescents’ risk of depression varies by gender (Salk et al., 2017), grade, and region (rural or urban) differences exist (Li et al., 2019). A systematic evaluation study found that sleep deprivation increases the risk of depressive mood, while exercise decreases this risk (Pemberton and Fuller Tyszkiewicz, 2016).In a meta-analysis, it was found that females, higher grades, and rural adolescents exhibited a higher risk of developing depression (Li et al., 2019).

In recent years, research on the impact of physical activity on depression has gained increasing popularity (Pascoe and Parker, 2019; Laird et al., 2023). Several cross-sectional studies have demonstrated a correlation between poor physical activity and depression prevalence, particularly in low- and middle-income nations (Stubbs et al., 2016; Mc Dowell et al., 2018). Multiple prospective observational and cohort studies suggest that activity of any intensity can prevent depression, with greater physical activity associated with a lower likelihood of depression (Mammen and Faulkner, 2013; Schuch et al., 2018; Guo et al., 2022). Moderate to strenuous physical activity reduces the risk of psychological problems and suicidal ideation by reducing academic stress and increasing life satisfaction (Liu et al., 2020).

Current research consistently indicates that physical activity can complement standard treatments for individual depression, often improving depressive symptoms similarly to antidepressant medications and psychotherapy (Josefsson et al., 2014; Rebar et al., 2015). Adolescents who participate in aerobic exercise can significantly reduce their levels of stress, tension, or anxiety, thereby helping to alleviate negative emotions and prevent mental health issues (Hilyer et al., 1982; Khalsa et al., 2012; Edwards et al., 2017). Moderate to vigorous physical activity is closely associated with a reduced risk of depression, contributing to its prevention (McDowell et al., 2018). In treating and preventing adolescent depression, physical activity emerges as a more practical and acceptable option compared to psychotherapy and medication (Rohden et al., 2017; Hetrick et al., 2021).

The effect of physical activity or exercise on depression varies with different frequencies and durations (Wang et al., 2022). Most studies have found that there is a positive dose–response relationships between exercise duration/frequency and depressive symptoms improvement (Roeh et al., 2019). Adhering to current WHO guidelines for physical activity, which recommend 150–300 min per week (or a minimum of 30 min per day for 5 days per week) of moderate-intensity aerobic activity, was associated with a 28% lower risk of major depression (McDowell et al., 2018). In studies of adults, exercise frequency was also found to be negatively associated with depressive symptoms and overall well-being (Hassmén et al., 2000). Another study showed that there is an U-shaped relationship between the physical activity levels and mental health symptom relief in adolescents (Shimura et al., 2023).

It is important to emphasize that, although exercise is often used synonymously with physical activity in reports, their definitions should not be conflated (Budde et al., 2016). Physical activity is defined as any body movement generated by skeletal muscles, characterized by its modality, frequency, intensity, duration, and context of practice, with exercise being a subset of it (Budde et al., 2016). In this study, we focus on physical activity, providing evidence from Chinese adolescents^1^ on the impact of times and hours of activity on depression. Additionally, by incorporating quadratic terms in our regression model, we further explore the nonlinear effects of physical activity on depression.

## 2 Materials and methods

### 2.1 Data collection

This study uses a cross-sectional dataset collected in September 2022 from Ruyang County, Henan Province. All participating students, their parents, and school teachers and principals were fully informed about the survey’s purpose and agreed to participate.

The sample was collected using a multi-stage, stratified, random sampling procedure. It covers 31 primary and secondary schools across 11 towns in Ruyang County. The specific steps are as follows: first, one middle school and one primary school were randomly selected from each town, resulting in 11 primary schools and 10 middle schools (there is no public middle school in Sanlitun Town). Second, to ensure a high follow-up rate, fourth and fifth graders were selected from primary schools, and seventh and eighth graders were selected from middle schools. Finally, two fourth-grade and two fifth-grade classes were randomly selected from each primary school, and two seventh-grade and two eighth-grade classes were randomly selected from each middle school. All students in the selected classes were included in the sample.

The survey included student questionnaires, parent questionnaires, and school questionnaires. Students completed the questionnaires within a limited time on-site, with instructions from the surveyors. Parent questionnaires were conducted using Wenjuanxing (Wenjuanxing is an app designed as an online survey platform to assist users in creating, sharing, and analyzing survey questionnaires). School questionnaires were completed through interviews between surveyors and school principals. After ensuring the completeness and validity of the questionnaires and excluding missing data and outliers, 5,629 observations were retained.

### 2.2 Variable selection

#### 2.2.1 Dependent variables

The dependent variable in this study is calculated based on the Center for Epidemiologic Studies Depression Scale (CES-D). The depression measurement properties of the CES-D have been empirically tested and can assess samples of children and adolescents (The BELLA Study Group et al., 2008). The scale demonstrates adequate internal consistency, good content validity, and appropriate convergent and discriminant validity, making it a reliable self-report tool for detecting depression in Chinese children and adolescents (William Li et al., 2010; Dou et al., 2021; Zhu et al., 2021).

The CES-D is currently the only internationally disseminated test that uses a multidimensional approach to measure depressiveness in children and adolescents (The BELLA Study Group et al., 2008). The CES-D includes 20 standardized items for assessing depressive symptoms (see Table 1). These items encompass four factors: depressive affect, positive affect, somatic symptoms, and interpersonal relations. Respondents are asked to report the frequency of specific emotions they experienced over the past week, with scores ranging from 0 to 3. A score of “0” represents “rarely or less than 1 day,” “1” represents “some of the time or 1–2 days,” “2” indicates “a moderate amount of the time or 3–4 days,” and “3” indicates “most or all of the time or 5–7 days.” The total score for all items provides a measure of depressive tendency, ranging from 0 to 60, with higher scores indicating more severe depression (Zhu et al., 2021). Therefore, we selected the depression score as the primary measure of adolescent depression, calculated by summing the items on the CES-D scale, as shown in Table 1.

**Table 1:** CES-D scale items and factors.

	Scale items	Factors
1	I was bothered by things usually do not bother me	Depressed mood
2	My appetite was poor	Somatic complaints
3	I felt that I could not shake off the blues even with help from my family or friends	Depressed mood
4	I felt I was just as good as others	Positive mood
5	I had trouble keeping my mind on what I was doing	Somatic complaints
6	I felt depressed	Depressed mood
7	I felt that everything I did was an effort	Somatic complaints
8	I felt hopeful about the future	Positive mood
9	I thought my life had been a failure	Depressed mood
10	I was fearful	Depressed mood
11	My sleep was restless	Somatic complaints
12	I was happy	Positive mood
13	I talked less than usual	Somatic complaints
14	I felt lonely	Depressed mood
15	People were unfriendly	Interpersonal
16	I enjoyed life	Positive mood
17	I had crying spells	Depressed mood
18	I felt sad	Depressed mood
19	I felt that people disliked me	Interpersonal
20	I could not get “going”	Somatic complaints

CES-D scale can only be used for screening depressive and cannot be used for diagnosis (Perreira et al., 2005).

Based on relevant international and Chinese studies, this paper adopts the commonly used cutoff score of 16 to determine the presence of depressive tendencies (McKhann et al., 1997; Blumenthal et al., 2003; Jiang et al., 2019; Dou et al., 2021). In this study, depression is used as another dependent variable, when the score of depression≤15 indicates no depression, while the score of depression ≥16 indicates the presence of depression (Blumenthal et al., 2003). It is important to emphasize that the CES-D scale’s classification of depression serves as a screening result and should not be used as a clinical diagnosis (Perreira et al., 2005).

#### 2.2.2 Independent variables

Physical activity is our key independent variable.^2^ We measured physical activity in terms of times of activity variables and hours of activity variables. The times of activity is measured by “How many times per week do you participate in physical activities (including badminton, soccer, long-distance running, etc.)?.” The hours of activity is obtained by multiplying “On average, how long (in hours) do you participate in physical activity each time?” multiplied by times of activity, which represents the total amount of time a youth spends in physical activity during the week.

#### 2.2.3 Control variables

Previous empirical studies have shown that many demographic variables are related to adolescent depression (Zhu et al., 2021; Zou et al., 2023), so we selected some demographic variables to control the variables. Individual characteristics control variables are as follows: gender, grade, household registration, the level of education of the father and mother. In addition, several studies have found that sleep deprivation increases the risk of depressive (Ojio et al., 2016; Li et al., 2020). Thus, we included the hours of sleep as a control variable to more objectively reflect the effect of physical activity on depression levels in adolescents. Table 2 shows all the variables and definitions of variables selected.

**Table 2:** Definition of variables and descriptive results (*n* = 5,629).

Variable	Definition	Mean ± SD	min	max
Dependent variables				
Depression score	Denotes the total depression score calculated from the CES_D scale.	14.608 ± 8.785	0	56
Depression	When score of the CES_D scale less than 16 is no depression, depression =0, 1 otherwise.	0.345	0	1
Independent variables				
Times of activity	The times of activity times per week	2.208 ± 1.596	0	10
Hours of activity	The hours of activity per week	2.710 ± 2.974	0	20
Control Variables				
Hours of sleep	The hours of sleep per day	9.739 ± 1.572	6	15
Boy	boy = 1, girl = 0	0.514	0	1
Grade	grade(four, five, seven, eight)	5.799 ± 1.571	4	8
Rural	Whether household registration is in a rural area, 1 for yes, 0 otherwise	0.834	0	1
Edufather	Father’s years of education	9.970 ± 2.584	0	16
Edumother	Mother’s years of education	9.834 ± 2.951	0	16

Mean indicates the mean value. SD denotes standard deviation.

#### 2.2.4 Descriptive statistical analysis of variables

The depression level, physical activity and demographic characteristics of the sample of 5,629 Chinese adolescents are shown in Table 2. Table 2 shows: the average depression score of adolescents was 14.608 (SD = 8.785); about 34.5% of adolescents exhibit varying degrees of depressive tendencies; adolescents engaged in about 2.208 times physical activities per week (SD = 1.596), and the hours of physical activity per week was 2.710 h (SD = 2.974), which is much lower than the number of hours recommended by the World Health Organization; and the average hours of sleep was more than 9 h (Mean = 9.739, SD = 1.572), which is within the range recommended by the National Sleep Foundation and the Canadian recommendations of the 24-h physical activity guidelines for children and adolescents (Tremblay et al., 2016; Mireku and Rodriguez, 2021); there was little gender difference, with 83.4% of the sample coming from rural areas and parents with almost 9 years of education.

### 2.3 Methods

We used ordinary least squares (OLS) to analyze the effect of physical activity on adolescent depression score (Equation 1) and probit modeling to analyze the effect of physical activity on adolescent depression (Equation 2). In order to verify whether there is an U-shaped relationship between physical activity and depression, we used a quantile regression model for the analysis.

In addition, we incorporated school fixed effects into the model, aiding in controlling for unobserved school-related confounders such as school culture, instructional quality, and geographical location. In summary, the introduction of school fixed effects allows for a more precise reflection of the impact of physical activity on depression across various school environments.

The regression model established is as follows:


(1) $${\mathrm{depression score}}_{i}=\alpha_{0}+\alpha_{1}X_{i}+\lambda Z+S{\mathrm{cool}}_{j}+\varepsilon_{i}$$

(2) $${\mathrm{depression}}_{i}=\beta_{0}+\beta_{1}X_{i}+\sigma Z+S{\mathrm{cool}}_{j}+\mu_{i}$$

Where X_i_ is the independent variable, including times of activity and hours of activity; Z is the group of control variable (see Table 2); School_j_ is the school fixed effects.

To examine whether there is a U-shaped relationship between physical activity and depression, we included the squared terms of times of activity and hours of activity in models (1, 2). By analyzing the coefficients of both the linear and squared terms, we can determine the optimal weekly times and hours of physical activity. Specifically, we calculate the optimal values by dividing the coefficient of the linear term by twice the negative coefficient of the squared term (Shimura et al., 2023).

## 3 Results

### 3.1 Regression results

Regression results for the effect of physical activity on adolescent depression scores are given in Table 3. The results in Table 3 show that using the OLS method, times of activity was significantly negatively correlated with adolescent depression score, whether or not hours of sleep were controlled for (*p* < 0.01). However, when controlling for hours of sleep, hours of activity was negatively correlated with adolescent depression score, but this was not statistically significant (β = −0.053, *p* = 0.172). The results in column 1 of Table 3 indicate that after controlling for hours of sleep and other demographic characteristics, an increase in physical activity by one time per week was associated with a mean decrease of 0.354 points in depression scores (β = −0.354, *p* < 0.01).

**Table 3:** The effect of physical activity on depression score.

Variable	Depression score			
Times of activity	−0.354^***^	−0.391^***^		
	(0.075)	(0.068)		
Hours of activity			−0.041	−0.055
			(0.044)	(0.044)
Hours of sleep	−1.081^***^		−1.093^***^	
	(0.092)		(0.092)	
Boy	−0.414	−0.309	−0.455	−0.349
	(0.305)	(0.319)	(0.309)	(0.325)
Grade	−0.533	−0.473	−0.601^*^	−0.547
	(0.338)	(0.374)	(0.344)	(0.382)
Rural	−0.711^*^	−0.802^*^	−0.691^*^	−0.784^*^
	(0.356)	(0.432)	(0.348)	(0.424)
Edufather	−0.135^**^	−0.148^**^	−0.144^**^	−0.157^***^
	(0.056)	(0.056)	(0.056)	(0.056)
Edumother	−0.079^*^	−0.091^*^	−0.082^*^	−0.094^**^
	(0.044)	(0.045)	(0.043)	(0.044)
School fixed effects	Yes	Yes	Yes	Yes
Constant	31.396^***^	20.454^***^	31.287^***^	20.217^***^
	(1.949)	(1.802)	(1.981)	(1.828)
Observations	5,629	5,629	5,629	5,629
R-squared	0.067	0.035	0.063	0.031

Numbers in parentheses are robust standard errors clustered to the school level. “Yes” indicates that this factor was controlled for.

*** *p* < 0.01.

** *p* < 0.05.

* *p* < 0.1.

The marginal effect of physical activity on the depression in adolescents using a probit regression model (see Table 4). The depression of adolescents decreased by an average of 1% when the times of activity increased by once a week, as shown in column 1 of Table 4. Our results indicate that times of activity significantly alleviated adolescent depression, whereas the relationship between the hours of activity and adolescent depression was not significant in either Table 3 or Table 4 without accounting for hours of sleep.

**Table 4:** The marginal effects of physical activity on depression.

Variable	Depression			
Times of activity	−0.011^**^	−0.012^***^		
	(0.004)	(0.004)		
Hours of activity			−0.001	−0.001
			(0.002)	(0.002)
Hours of sleep	−0.047^***^		−0.047^***^	
	(0.004)		(0.004)	
Boy	−0.022	−0.017	−0.023^*^	−0.019
	(0.016)	(0.017)	(0.016)	(0.017)
Grade	−0.023	−0.020	−0.025	−0.023
	(0.017)	(0.018)	(0.017)	(0.019)
Rural	−0.027	−0.003	−0.025	−0.029
	(0.017)	(0.020)	(0.016)	(0.019)
Edufather	−0.005^*^	−0.006^**^	−0.006^**^	−0.006^**^
	(0.003)	(0.003)	(0.003)	(0.003)
Edumother	−0.003	−0.003	−0.003	−0.004
	(0.003)	(0.003)	(0.003)	0.003
School fixed effects	Yes	Yes	Yes	Yes
Observations	5,629	5,629	5,629	5,629

Numbers in parentheses are robust standard errors clustered to the school level. “Yes” indicates that this factor was controlled for.

*** *p* < 0.01.

** *p* < 0.05.

* *p* < 0.1.

**Figure 1:**
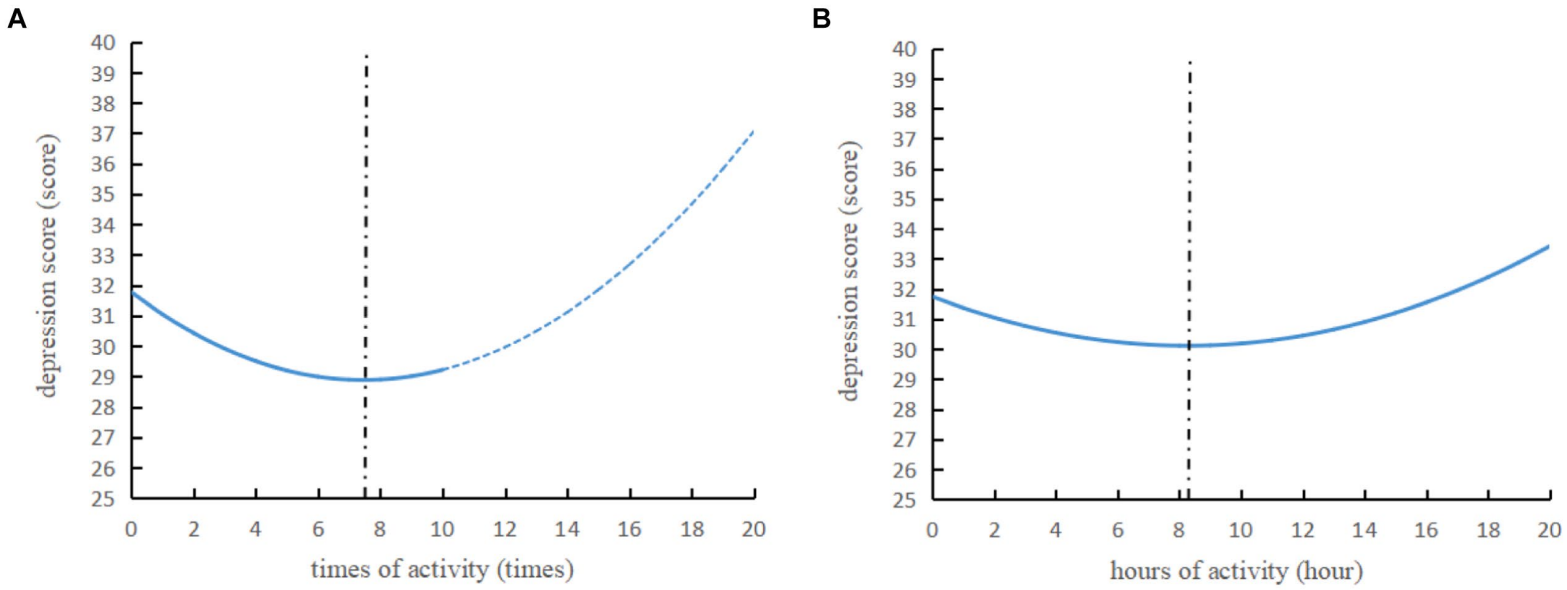
The optimal level of physical activity calculated from the results of the quadratic equation The curve formula in panel **(A)** is depression score = −0.775 × (times of activity) + 0.052 × (times of activity2) + 31.768, and the curve formula in panel **(B)** is depression score = −0.396 × (hours of activity) + 0.024 × (hours of activity2) + 31.740. The values of x range from 0 to 10 times. To better illustrate the U-shaped curve, values greater than 10 are shown with a blue dashed line. According to the quadratic function formula, the optimal activity times in panel **(A)** are calculated to be 7.452 times, and the optimal activity duration in panel **(B)** is calculated to be 8.250 h, indicated by a black vertical dashed line. Times of activity2 and hours of activity2 are squared terms of times of activity and hours of activity, respectively.

The regression results after adding the square term are shown in Table 5. The results in Table 5 indicate that there is a U-shaped relationship between physical activity and adolescent depression, suggesting that physical activity is not as good as more. Based on the formula for calculating the optimal weekly times and hours of physical activity (Shimura et al., 2023), we determined that the optimal times is 7.300–7.452 times per week (−0.775/(2 × 0.052) = 7.452, −0.073/(2 × 0.005) = 7.300, Table 5, Columns 1 and 3, Figure 1). The optimal hours is 7.833–8.250 h per week (−0.396/(2 × 0.024) = 8.250, −0.047/(2 × 0.003) = 7.833, Table 5, Columns 2 and 4). Thus, our results suggest that the 7–8 times per week and 7–9 h of activity per week were found to be associated with optimal improvement in depression.

**Table 5:** Regression results of non-linear effects of physical activity on depression.

Variable	Depression score	Depression	Depression score	Depression
Times of activity	−0.775^***^	−0.073^**^		
	(0.216)	(0.035)		
Times of activity2	0.052^**^	0.005		
	(0.024)	(0.004)		
Hours of activity			−0.396^***^	−0.047^***^
			(0.120)	(0.017)
Hours of activity2			0.024^***^	0.003^***^
			(0.006)	(0.001)
Control variables	Yes	Yes	Yes	Yes
School fixed effects	Yes	Yes	Yes	Yes
Constant	31.768^***^	1.672^***^	31.740^***^	1.679^***^
	(1.957)	(0.306)	(1.950)	(0.304)
Observations	5,629	5,629	5,629	5,629

Numbers in parentheses are robust standard errors clustered to the school level. Times of activity2 and hours of activity2 are squared terms of times of activity and hours of activity, respectively. “Yes” indicates that this factor was controlled for. ^*^p < 0.1.

*** *p* < 0.01.

** *p* < 0.05.

### 3.2 Heterogeneity analysis

Some studies have shown that there are significant differences in depressive symptoms among adolescents by gender, grade, and region (Lee et al., 2013; Wesselhoeft et al., 2013; Salk et al., 2017; Li et al., 2019). Therefore, we analyzed the effect of times of activity on depression score and depression across gender, grade, and region (see Tables 6, 7 for results). The regression coefficients in Table 6 show that there is a significant mitigating effect of times of activity on adolescent depression across gender, grade level, and region, and that times of activity has a greater mitigating effect on adolescent depression in boys, upper grades (junior high school students), and urban areas. The results in Table 7 show that times of activity has a significant effect on whether or not adolescents are depressed in boys and upper grades (junior high school students), with a greater mitigating effect on whether or not adolescents are depressed in urban areas.

**Table 6:** Results of the heterogeneity analysis of times of activity on depression score.

Variable	Depression score					
	Boy	Girl	Elementary	Junior	Rural	Urban
Times of activity	−0.399^***^	−0.262^**^	−0.348^***^	−0.376^**^	−0.351^***^	−0.401^***^
	(0.099)	(0.124)	(0.088)	(0.132)	(0.091)	(0.119)
Control variables	Yes	Yes	Yes	Yes	Yes	Yes
School fixed effects	Yes	Yes	Yes	Yes	Yes	Yes
Constant	29.270^***^	33.025^***^	26.558^***^	30.180^***^	31.704^***^	32.201^***^
	(2.210)	(2.969)	(1.470)	(2.193)	(2.127)	(3.730)
Observations	2,893	2,736	3,219	2,410	4,697	932

Numbers in parentheses are robust standard errors clustered to the school level. Elementary denotes lower elementary students; junior denotes upper junior high school students. “Yes” indicates that this factor was controlled for. ^*^p < 0.1.

*** *p* < 0.01.

** *p* < 0.05.

**Table 7:** Results of the heterogeneity analysis of times of activity on depression.

Variable	Depression					
	Boy	Girl	Elementary	Junior	Rural	Urban
Times of activity	−0.046^**^	−0.009	−0.020	−0.047^**^	−0.031^**^	−0.033^*^
	(0.019)	(0.017)	(0.013)	(0.024)	(0.015)	(0.018)
Control variables	Yes	Yes	Yes	Yes	Yes	Yes
School fixed effects	Yes	Yes	Yes	Yes	Yes	Yes
Constant	1.710^***^	1.475^***^	1.070^***^	1.305^***^	1.626^***^	2.277^***^
	(0.392)	(0.361)	(0.182)	(0.331)	(0.320)	(0.682)
Observations	2,893	2,736	3,219	2,410	4,697	932

Numbers in parentheses are robust standard errors clustered to the school level. Elementary denotes lower elementary students; junior denotes upper junior high school students. “Yes” indicates that this factor was controlled for.

*** *p* < 0.01.

** *p* < 0.05.

* *p* < 0.1.

## 4 Discussion

We found that the times of physical activity improved adolescent depression, while the relationship between the hours of physical activity and depression was not significant. This is similar to the results of Roeh et al. (2019) and Mead et al. (2009). It probably due to the fact that for adolescents, the shorter the effective duration of physical activity, the more likely to increase adolescents’ motivation to participate in physical activity (Downs et al., 2013). Decreased energy levels are a characteristic symptom of depressed individuals for whom long-term sustained exercise may be too demanding. Although this study has similar results to the aforementioned studies, there are two key differences. First, our focus is on physical activity rather than exercise, and the two have different meanings (Budde et al., 2016). Second, our study differs from Roeh et al. (2019) in the definition and measurement of variables. In Roeh et al. (2019), the duration of exercise refers to the length of each exercise session, whereas we measure the total hours of activity over a week for adolescents (Roeh et al., 2019). Our results suggest that increasing the frequency of physical activity, rather than the total number of hours, is more effective in improving adolescent depression, which is consistent with the special emphasis in the “Healthy China 2030 Action Plan,” which emphasizes that school-age children and adolescents should participate in no less than 60 min of in-school physical activity per day (as opposed to the total number of hours per week), and recommends that they should engage in moderate-intensity activity at least three times per week.^3^ Approximately 170 million children and adolescents in China have inadequate levels of physical activity (Chen et al., 2020). This study therefore adds to the consensus that physical activity may have benefits for adolescent depression, and that increased physical activity has a very important role to play in the healthy growth and lifelong well-being of adolescents.

In addition, we found an U-shaped relationship between physical activity and depression in adolescents, suggesting that higher physical activity frequency is not better. This is similar to the findings of Chen et al. (2020) and Shimura et al. (2023), indicating that physical activity is not linearly associated with depression improvement (Chen et al., 2020; Shimura et al., 2023). In addition, several studies have confirmed that more activity is not always better, and that too much or too long and out of the optimal range of physical activity is detrimental to depression, i.e., optimal frequency of physical activity or duration of activity should be maintained to improve neuroticism and enhance resilience thereby alleviating or protecting against depressive symptoms (Kuwahara et al., 2015; Chekroud et al., 2018; Kim et al., 2018; Shimura et al., 2023). We have found that while increased frequency of physical activity is associated with improved depression in adolescents, it may be counter-productive when exceeding the optimal range of physical activity of approximately seven to eight times per week and 7 to 9 h of activity per week, i.e., too frequent or too prolonged physical activity. To date, no guidelines or optimal goals have been developed for levels of physical activity that are effective in preventing depression. We must emphasize that we can only offer possible interpretations of these results. The findings of this study may help set the standard for further research.

The analysis of heterogeneity in Tables 6, 7 shows that increasing the times of physical activity has a significant ameliorating effect on the level of depression among adolescents of different genders, grades, and regions, and that there is some variability in the effect on whether adolescents are depressed or not. The differences between Tables 6, 7 may stem from differences in sample characteristics, and variable selection. It may be due to the fact that boys, upper grades and urban adolescents engage in more strenuous physical activity and therefore increase endorphin secretion, better adjusting the psyche thus reducing the level of depression (Bender et al., 2007; Goldfield et al., 2011). Therefore, we are going to need to take these factors into account to interpret these differences with caution.

Our study still has some limitations. First, the measurement of the dependent variable in this paper may be affected by individual subjective factors and have response bias, and cannot be used for accurate individual depression diagnosis, which can be combined with other clinical assessment tools and doctors’ diagnosis to make a comprehensive judgment in the future study, so as to improve the accuracy of the measurement of depression in adolescents. Diagnosis to synthesize the judgment, thus improving the precision of measuring depression in adolescents. Second, due to the limited nature of the data, there were no data on activity intensity. Therefore, we only included times and hours of activity as a key influencing factor in this study, and future research could include more other influencing factors. Finally, cross-sectional data only allowed us to make possible interpretations of the causal relationship between physical activity and depression. Therefore, it is suggested that it could be supplemented as panel data in further future studies to assess the causal effect of physical activity on depression in adolescents.

## 5 Conclusion

We explored the effects of physical activity on depression in a group of Chinese adolescents, using data from 5,629 cross-sectional studies. The results of the study reflect that increasing the times of activity is a positive factor in improving adolescent depression, but increasing the hours of physical activity was not significantly associated with adolescent depression. Therefore, we believe that adolescents should be encouraged to encourage the formation of a regular physical activity habit and increase the frequency of activity, which should preferably be spread out over several days per week rather than concentrated in 1 or 2 days.

Our results also illustrated that there is a U-shaped relationship between times of activity, hours of activity and depression. Therefore, we recommend that adolescents engage in physical activity 7 to 8 times and 7 to 9 h per week. Activity frequency and hours in this range are associated with improved depression, so moderate activity is key.

We found differences in the effects of physical activity on depression among adolescents of different genders, grades, and regions. Therefore, it is advisable to pay attention to the feedback and experiences of individuals and different groups and adjust the frequency and hours of activities to accommodate different needs and preferences. In addition, we can find that sleep has a significant mitigating effect on adolescent depression in all regression results. Therefore, it is important to recognize the influence of other factors (e.g., sleep duration) on depression in addition to physical activity. Incorporating these factors into intervention strategies can contribute to an integrated approach to improving adolescents’ depression. Our findings and recommendations provide valuable guidance for parents, educators, and community organizations on effectively using physical activity to reduce the probability of depression in adolescents.

## Data availability statement

The data analyzed in this study is subject to the following licenses/restrictions: the data is considered proprietary or under intellectual property rights, which restrict its distribution without permission. Requests to access these datasets should be directed to zhangyingcau@126.com.

## Ethics statement

The studies involving humans were approved by China Agricultural University Institutional Review Board. The studies were conducted in accordance with the local legislation and institutional requirements. The participants provided their written informed consent to participate in this study.

## Author contributions

HC: Data curation, Formal analysis, Methodology, Validation, Writing – original draft, Writing – review & editing. ML: Data curation, Formal analysis, Methodology, Validation, Writing – original draft, Writing – review & editing. WZ: Data curation, Formal analysis, Investigation, Writing – original draft. HW: Writing – original draft, Data curation, Formal analysis. YZ: Conceptualization, Data curation, Formal analysis, Methodology, Resources, Writing – review & editing. SL: Data curation, Formal analysis, Writing – review & editing, Conceptualization, Methodology, Resources.

## Footnotes

^1^The World Health Organization defines adolescence as the period between the ages of 10 and 19 years, and this paper follows this definition, with the age of the sample falling within that age range and therefore defined as adolescents. For more details, see: https://www.who.int/topics/adolescent_health/en/.

^2^The WHO definition of physical activity is used in this paper. WHO defines physical activity as any bodily movement produced by skeletal muscles that requires energy expenditure. See: https://www.who.int/news-room/fact-sheets/detail/physical-activity.

^3^Available: http://www.gov.cn/zhengce/2016-10/25/content_5124174.htm
